# Recent advances in fecal microbiota transplantation for *Clostridium difficile* infection-associated diarrhea after kidney transplantation

**DOI:** 10.3389/frmbi.2024.1409967

**Published:** 2024-07-16

**Authors:** Yurong Li, Yaoyao Yang, Ning Yang, Qin Wu, Jinjin Yang, Jing Guo, Hongmei Zhang

**Affiliations:** ^1^ Department of Urology, Henan Provincial Key Medicine Laboratory of Nursing, Henan Provincial People’s Hospital, Zhengzhou University People’s Hospital, Henan University People’s Hospital, Zhengzhou, Henan, China; ^2^ Nursing Department, Henan Provincial Key Medicine Laboratory of Nursing, Henan Provincial People’s Hospital, Zhengzhou University People’s Hospital, Henan University People’s Hospital, Zhengzhou, Henan, China

**Keywords:** kidney transplantation, diarrhea, fecal microbiota transplantation, *clostridium difficile* infection, kidney disease

## Abstract

Kidney transplantation is considered to be the best treatment for end-stage renal disease. To reduce the incidence of rejection and improve the survival of recipients and kidney grafts, kidney transplant recipients must take immunosuppressive agents, and some patients require them for the rest of their lifetime. These treatment regimens can result in susceptibility to opportunistic infections and disrupt the intestinal microbiota, thereby leading to diarrhea, which causes water and electrolyte metabolism disorder, nutrient malabsorption, and instability in the blood concentrations of the immunosuppressive agents. Fluctuating blood concentration levels of these agents necessitate frequent laboratory monitoring and dose adjustments to avoid poor adherence and increase the risk of graft rejection. Furthermore, severe diarrhea can cause kidney transplant failure or death. *Clostridium difficile* infection (CDI) is the leading cause of diarrhea after renal transplantation. Traditional antibiotics can kill *C. difficile*; however, spores can remain in the gut. Disruption of the intestinal flora caused by antibiotherapy increases the risk of developing recurrent CDI (rCDI). Fecal microbiota transplantation (FMT) has been proven to be a safe and effective treatment for CDI and is recommended for rCDI owing to its convenient material acquisition method, high efficacy, and low incidence of adverse reactions. This review summarizes the recent progress in FMT for CDI-associated diarrhea after renal transplantation.

## 1 Introduction

Following the first successful kidney transplantation (KT) by Joseph Murray in 1954, KT became the preferred treatment for patients with end-stage kidney disease (Davor et al., 2020). According to statistics from the Global Observatory on Donation and Transplantation, 90,306 KTs were performed worldwide in 2017 (Cappadona et al., 2020). The Chinese Scientific Registry of Kidney Transplantation data showed that 11,030 KTs were performed in China in 2021, which is the second-highest number of cases among those of other countries (Pan et al., 2022). Following its introduction in the 1950s, KT became the most developed and common type of solid organ transplantation worldwide, especially with the development of immunosuppressants. Despite advances in surgical techniques and the use of new technologies, KT is still associated with various clinical and surgical complications owing to its high complexity and differences among patients.

Diarrhea is a common complication of KT. Severe diarrhea can affect the survival of transplanted kidneys. Diarrhea after KTs is closely associated with intestinal *Clostridium difficile* infections (CDIs) (Khoruts et al., 2009). *Clostridioides difficile* (CD) is a gram-positive endospore-producing anaerobe that can colonize the intestinal microbiome (Goudarzi et al., 2014). Disturbances in the intestinal microbiome increase patient vulnerability to CDI, which can lead to diarrheal diseases and pseudomembranous enteritis and be fatal (Riley and Kimura, 2018). CD is a major causative agent of nosocomial and antibiotic-associated infectious diarrhea (Britton and Young, 2014). To enhance the survival rates of both recipients and kidney grafts while minimizing rejection, it is imperative for KT patients to adhere to lifelong immunosuppressive therapy and long-term antibiotic treatment. However, these treatment regimens can lead to increased susceptibility to opportunistic infections and various gastrointestinal symptoms. Diarrhea is the most prevalent symptom, occurring in approximately 13–53% of patients (Aulagnon et al., 2014; Sonambekar et al., 2020). A survey conducted in the USA reported that CDI, norovirus infection, and cytomegalovirus infection were the main etiologies of diarrhea in KT recipients (Zhou et al., 2011). Currently, antibiotics remain the preferred treatment for CDI; however, conventional antibiotic therapy regimens frequently result in recurrent CDI (rCDI), with up to 45–65% of patients experiencing multiple relapses (McPake and Burnapp, 2009). Given the persistently high rates of recurrence, effective therapeutics are urgently needed for both primary and recurrent CDIs. Fecal microbiota transplantation (FMT) is highly effective in preventing and treating CDIs. It is an emerging therapeutic approach that restores host function and involves the transfer of healthy donor fecal microbiota to the gastrointestinal tracts of patients, and it has applications in both intestinal and extra-intestinal diseases (Zhu et al., 2018). FMT has been successfully employed for the treatment of various diseases, including CDI, inflammatory bowel disease, and irritable bowel syndrome (Zhu et al., 2020). Furthermore, it has been shown to effectively treat rCDI in humans and has a higher cure rate than standard antibiotic treatments (Le et al., 2018). Compared to metronidazole, vancomycin, and fidaxomicin, FMT is one the most cost-effective treatments for rCDI, with mean cure rates of 80–90% (Gianluca et al., 2018).

FMT originated in 4th century China (Yoshimatsu et al., 2021). Moreover, in the Lis Compendium of Materia Medica, more than 20 therapeutic approaches for treating diseases with feces have been recorded (Shizhen, 2011). In 2011, FMT was recommended as a treatment for rCDI in professional guidelines from the USA and UK (Borody and Khoruts, 2011). Subsequently, in 2013, the application of FMT for rCDI was advocated for by the National Institute for Clinical Excellence. Guidelines for treating CDI also recommend that FMT be considered if a third recurrence occurs after treatment with a pulsed vancomycin regimen (Surawicz et al., 2013). FMT restores the microbial community structure in the colon, which protects against CD colonization and suppresses the growth and production of disease-causing toxins. It has also been shown to be safe and feasible in patients who had undergone KT and were immunosuppressed, without increasing the incidence of infection, and no severe complications were observed (Pratik et al., 2018). This review summarized recent progress in the application of FMT for CDI-associated diarrhea after renal transplantation.

## 2 Research status of treatment methods for CDI

The Gram-positive, anaerobic, and spore-forming bacillus CD is the most common cause of nosocomial diarrhea worldwide. According to previous studies conducted in China, CDI has a pooled prevalence of 14.0% among hospitalized patients with diarrhea, and CD is the cause of approximately 19% of antibiotic-associated diarrhea cases (Xie et al., 2017). Metronidazole and vancomycin are the mainstays of CDI treatment (Khanna, 2021). With the aim of improving the overall cure rate of CDI, a novel therapeutic approach that includes FMT has gained recognition and has demonstrated good clinical efficacy (Khoruts et al., 2021). Below is an overview of the current status of emerging preventative and treatment methods for CDI.

### 2.1 Immunomodulatory treatments

#### 2.1.1 Vaccines

Vaccinations are an economical means of preventing CDI and the target population is adults at risk of CDI (Yang and Wang, 2022). Rees and Steiner (2018) found that gut microflora dysbiosis and insufficient levels of serum antibodies against CD toxins are associated with CD relapse, suggesting that TcdB and TcdA may be excellent immunogens for vaccine research and development (Tian et al., 2012). Additionally, nucleic acid-based vaccines for CD and non-toxic component vaccines targeting a surface-associated antigen of CD have achieved good results in animal trials (Feher et al., 2017). These vaccine candidates are currently undergoing clinical trials. Toxoid TcdA and TcdB protein vaccines, produced by Sanofi Pasteur, were the first vaccines to undergo human trials but were discontinued for failing to reduce the incidence of CDI in high-risk group (de Bruyn et al., 2020). Pfizer developed a CD recombinant bivalent toxin vaccine. According to the results of phase II clinical studies, it has a good safety profile and is highly immunogenic in adults aged 65–85 years (Nicholas et al., 2020). Phase III trials of these vaccines are ongoing (Nicholas et al., 2020). The French company Valneva developed a recombinant chimeric subunit vaccine (VLA84) for CD, which incorporates the receptor binding domain (RBD) of both TcdA and TcdB through a 12-amino acid adaptor sequence (Bezay et al., 2016). Phase II clinical trials demonstrated that the vaccine had exceptional safety, efficacy, and tolerability. Moreover, it elicited robust levels of active antibodies against RBD in individuals aged 50–64 years and >65 years (Riley et al., 2019). In addition to the aforementioned vaccine products that are currently in the clinical trial stage, a diverse range of vaccines are currently undergoing preclinical evaluation, which encompasses oral mucosal, DNA, polysaccharide, and polysaccharide-protein conjugate vaccines.

#### 2.1.2 Therapeutic antibodies

rCDI may be associated with a diminished humoral immune response in the host (Rees and Steiner, 2018). Therapeutic antibodies targeting CD that actively suppress the production of cytotoxins have played a crucial role in reducing the recurrence of CDI (Posteraro et al., 2018).

Actoxumab and Bezlotoxumab are two fully humanized monoclonal antibodies, developed by Merck, that target TcdA and TcdB, respectively. These antibodies can effectively neutralize toxins, prevent damage to intestinal epithelial cells, aid in the restoration of the normal intestinal microbiome, and ultimately contribute to preventing rCDI (Llafuerte-Galvez and Kelly, 2017). Given that monoclonal antibodies lack antibacterial activity, they should not be considered as a substitute for antibiotic therapy in the treatment of CDI. Their use should be limited to combination therapy with conventional antibiotics (such as metronidazole, vancomycin, or fedamycin) for patients aged 18 years and above (Yang and Wang, 2022). The safety and efficacy of Actoxumab and Bezlotoxumab in combination with antibiotics for the treatment of CDI were evaluated in two phase III clinical trials (MODIFY I and MODIFY II) conducted by Merck (Posteraro et al., 2018). According to the findings of the interim evaluation of MODIFY I, treatment in the Actoxumab group was prematurely terminated due to inadequate safety and efficacy outcomes. However, a combined analysis of data from the MODIFY I and II studies revealed that Bezlotuxumab not only significantly reduced the recurrence rate of CDI but also substantially improved the overall cure rate compared to the those in the placebo group (Wilcox et al., 2017). Based on these findings, Bezlotoxumab was subsequently approved by the US Food and Drug Administration (FDA) and the European Medicines Agency (EMA) for marketing, becoming the first monoclonal antibody used to treat CDI (Forster et al., 2018). A recent study showed that Bezlotuxumab combined with antibiotic therapy has potential for treating solid organ transplant recipients with elevated susceptibility to rCDI (Reigadas et al., 2021).

In recent years, emerging nanobodies (Nb) have demonstrated distinct advantages in the treatment of CDI. Yang et al. (2016) developed a novel quadrivalent bispecific Nb that effectively induced the production of highly potent antibodies against both TcdA and TcdB toxins in animal models of CDI. Furthermore, this nanobody exhibited an extended half-life of >1 month and provided complete protection against primary and recurrent CDI in mice. Additionally, Kroh et al. (2018) engineered an innovative humanized Nb that neutralizes diverse ribotypes of CD-produced TcdB.

Hyperimmune bovine colostrum (HBC) is a cost-effective therapeutic antibody for treating CDI, which is obtained through immunization of pregnant cows with specific toxins, spores, and antigens to stimulate the production of colostrum rich in specific IgG or secretory IgA. Sponseller et al. (2015) demonstrated that cows immunized with recombinant mutant proteins of TcdA and TcdB were able to secrete HBC that showed effective therapeutic efficacy for CDI.

### 2.2 Antibiotics

#### 2.2.1 Current state of clinical treatment

Vancomycin (Van) and metronidazole (Mtr) are the current first-line antibiotics for CDI treatment and are widely used to treat initial CDI (Yang and Wang, 2022). However, they are associated with certain drawbacks, such as antibiotic resistance, disruption of intestinal flora, and high CDI recurrence rates. Due to the emergence of Mtr-resistant CD strains resulting in treatment failure and the broad-spectrum eradication of intestinal flora by Mtr, which is detrimental to CDI treatment, Mtr has gradually been phased out as the primary choice for CD antibiotic therapy (Johnson et al., 2014). The most recent Infectious Disease Society of America (IDSA) and Society for Healthcare Epidemiology of America (SHEA) guidelines issued in 2018 no longer recommend Mtr for treatment of either severe or non-severe CDI (McDonald et al., 2018). However, in patients with mild CDI, Mtr demonstrates comparable therapeutic efficacy to that of Van. Therefore, Mtr can still be considered as a viable option for initial treatment of CDI (Liu et al., 2022).

The most recent antibiotic to be approved for CDI treatment in the U.S. was fidaxomicin in 2011 (Khanna and Gerding, 2019). Fidaxomicin (Fid), a narrow-spectrum macrolide antibiotic, has more specific and durable antibacterial effects than Mtr and Van. It not only exhibits a higher overall cure rate (Fid = 73%, Van = 62.9%) but also a reduced risk of recurrence (Fid = 3.3%, Van = 4%) (Giacobbe et al., 2022). Fid has no effect on Gram-negative bacteria, and the main mechanism of action is the prevention of transcription via inhibition of RNA polymerase (Zhanel et al., 2015). In 2021, IDSA issued new guidelines on CD treatment that recommended Fid as the first-line treatment option and Van as a secondary alternative. Mtr is only recommended in the absence of other treatment options (Meuwly and Chuard, 2022). However, the high cost of Fid greatly limits its usage; thus, there is a need for the development of more economical and effective narrow-spectrum antibiotics (Yang and Wang, 2022).

#### 2.2.2 Novel antibiotics

Currently, a range of novel antibiotics are being evaluated in clinical trials, including ridinilazole, cadazolid, surotomycin, and LFF571. Cadazolid (Cdz, formerly known as ACT-179811) is a novel oxazolidinone antibiotic that is superior to Van and Mtr in inhibiting bacterial protein and toxin synthesis against a variety of clinical CD strains, with a 24-h bacterial killing rate of up to 99.9% (Rashid et al., 2013; Locher et al., 2014b). Macromolecular labeling experiments confirmed that the bactericidal mechanism of Cdz mainly involves the inhibition of protein synthesis (Locher et al., 2014a). Cdz was safe and well-tolerated but the primary endpoint of non-inferiority to Van in the clinical cure rate was not achieved in one of the two phase III trials (Gerding et al., 2019). Therefore, further commercial development of Cdz for CDI treatment has been suspended. Ridinilazole (Rdz, formerly known as SMT19969) is a new narrow-spectrum, non-absorbable antibiotic with high selective antibacterial activity against CD and a low drug resistance rate (Vickers et al., 2011). Due to its ability to reduce the recurrence of CDI, Rdz stands out among new drugs. Additionally, Rdz has been granted Fast Track designation by the FDA (Carlson et al., 2019). Studies utilizing high-resolution microscopy have indicated that Rdz may exert antibacterial effects by affecting cell division pathways (Basseres et al., 2016). To date, two phase II trials on Rdz have been conducted, and phase III trials are in planning (Carlson et al., 2019). Surotomycin (Sur, formerly known as CB-183, 315), a novel lipopeptide antibiotic developed by Merck, disrupts the cellular membrane of CD, thereby exerting a bactericidal effect (Locher et al., 2014a). However, Sur demonstrated inferior efficacy to that of Van in terms of a sustained cure rate and reduced CDI recurrence in the phase III clinical trial. Consequently, the development of Sur has been halted (Khanna and Gerding, 2019). LFF571, a semisynthetic thiopeptide inhibitor of bacterial protein synthesis, similar to GE2270A, interacts with the bacterial elongation factor Tu (Lyn et al., 2022). It exhibited remarkable antibacterial efficacy against CD and other Gram-positive anaerobic bacteria but demonstrated negligible antibacterial activity against anaerobic Gram-negative bacteria (Citron et al., 2012). In Phase II clinical trials, LFF571 exhibited a higher clinical cure rate than that of Van (90.6% versus 78.3%), with lower effective doses and recurrence rates (Mullane et al., 2019).

#### 2.2.3 Strategy for the utilization of antibiotics

Antibiotic therapy remains the first choice therapy for treating CDI, and the antibiotic should be chosen based on guidelines and the severity of the disease. The American College of Gastroenterology (ACG) recommends oral administration of 125 mg Van four times daily for 10 days, or oral administration of 200 mg Fid twice daily for 10 days, or oral administration of 500 mg Mtr three times daily for 10 days for patients with non-severe primary CDI (Kelly et al., 2021). For patients with severe CDI presenting with white blood cell counts of ≥15000 cells/ml or serum creatinine levels >1.5mg/dL, initial treatment should consist of a 10-day course of oral Van or Fid therapy, and Mtr is not recommended. In cases of fulminant CDI characterized by shock, intestinal obstruction, or megacolon, oral Van therapy is recommended and intravenous Mtr combination therapy can be considered if necessary. Additionally, in the event of complete intestinal obstruction, the recommendation is rectal retention enema with 500 mg Van in 100 ml saline every 6 hours (Ahmed and Kuo, 2020). For patients with rCDI, it is recommended to orally administer 125 mg Van four times daily for 14 days or 200 mg Fid twice daily after the first recurrence. The treatment duration should be 10 days. In cases of second or multiple recurrences, a high-dose Van pulse regimen, fecal microbiota transplantation, or 200 mg Fid twice daily for 10 days are recommended (Lyn et al., 2022).

### 2.3 Microbiome-therapy

The main methods for improving dysbiosis of the intestinal flora include FMT, the oral administration of the non-toxigenic CD strain M3 (NTCD-M3), and probiotics. FMT, which restores the normal composition and function of gut microbiota, is a strong therapeutic option for rCDI, with success rates of 80–90% (Gianluca et al., 2018). Wang et al. (2019) demonstrated in a randomized controlled trial that 81% of patients with CDI were relieved of symptoms after undergoing FMT through the nasoduodenal tube, and the overall therapeutic effect was significantly better than that of clinical antibiotic therapies, such as Van. According to Zou et al. (2020), FMT effectively treats newly diagnosed, recurrent, and refractory CDI by restoring colonization resistance and host immunity to CDI. Future research should focus on methods of precise regulation of the intestinal flora and personalizing FMT for different patients and situations. NTCD-M3 lacks toxin-producing genes and does not cause CDI in the gut. Upon entering the intestinal tract, NTCD-M3 competitively inhibits the binding of toxigenic CD to its receptor and prevents further colonization by toxigenic strains. However, this treatment raises potential safety concerns, such as the risk of recombination of TcdA and TcdB encoding genes between the residual toxigenic and nontoxigenic strains (Yang and Wang, 2022). Probiotics (Mainly including Lactobacillus and bifidobacterium) are live, non-pathogenic bacteria capable of colonizing the gut. They can through the following several ways to have beneficial effects on host: (1) regulating intestinal flora and inhibit the growth of pathogenic microorganisms breeding. (2) Enhancing the barrier function of intestinal epithelium. (3) Regulate the body’s immune response (Valdes et al., 2018). Studies have shown that probiotics have good effects in the treatment of idiopathic CDI (Yang and Wang, 2022).

At present, several phages that can lyse CD have been identified from the human gut. Nale et al. (2016) conducted a study comparing the efficacy of seven different phages in lysing CD, and the results showed that either alone or in combination, these phages were able to inhibit the formation of CD biofilm, disrupt pre-formed biofilms, and ultimately lead to the dissolution of CD. Hence, Phage therapy is expected to become an effective treatment for CDI.

## 3 Mechanisms of FMT in the treatment of CDI

### 3.1 Restoration of the richness and diversity of intestinal flora

Human intestinal flora comprise a complex and diverse community of commensal microorganisms. Under healthy conditions, human intestinal flora can resist CD colonization and provide a protective mucosal barrier to prevent CDI. Lin and Chen (2014) found that the diversity of the intestinal flora in patients with CDI decreased significantly, particularly the abundance of *Bacteroidetes* and *Firmicutes*. *Firmicutes* and *Bacteroidetes* are crucial for maintaining intestinal microecological homeostasis. *Firmicutes* produce large amounts of short-chain fatty acids (SCFAs). SCFAs perform antibacterial and anti-inflammatory functions, maintain intestinal epithelial barrier integrity, and regulate intestinal immunity, which can contribute to resisting colonization by pathogens such as CD. *Bacteroidetes* can inhibit the proliferation of CD in the intestinal cavity. In patients with rCDI who received FMT, the diversity of the intestinal flora increased 16 weeks after treatment. After multiple FMTs, the intestinal flora abundances were similar to those in healthy donors. The levels of *Firmicutes* and *Bacteroidetes* increased, while that of Proteus decreased, and the normal flora structure was maintained. The regulation of *Firmicutes* and *Bacteroidetes* levels is essential in maintaining a normal intestinal microbiome (Weingarden et al., 2015).

### 3.2 Restoration of bile acid metabolism levels

The levels of different bile salts in the intestines can affect CD colonization by directly regulating its germination and growth. Bile acids play important roles in the early stages of bacterial proliferation by inducing cortical degeneration, calcium release, and spore rehydration. Some bile acids promote the proliferation of CD spores, but the bile acid chenodeoxycholic acid inhibits it (Sorg and Sonenshein, 2010). Moreover, antibiotics cause changes in bile acid levels that support CD germination and growth. Patients with CDI have higher primary bile acid levels and lower secondary bile acid levels than those of the general population (Allegretti et al., 2016). A previous study found that FMT restored the metabolism of secondary bile acids in the intestinal tract of patients to a similar level to that in the donors. Additionally, the secondary bile salt levels increased after transplantation (Weingarden et al., 2014). Furthermore, before FMT, the bile acid composition in the gastrointestinal tracts of patients with rCDI can promote the proliferation of CD spores. In contrast, after treatment, the bile acid composition in patients can play an inhibitory role on CD spore formation (Weingarden et al., 2016).

### 3.3 Competition for intestinal nutrients

Intestinal flora interfere with the synthesis of toxic factors or directly cause kill CD by competing with CD for nutrients (Khoruts and Sadowsky, 2016). CDI mouse models treated with antibiotics showed increased levels of carbohydrates, sialic acid, and succinate in their intestines, which promoted the growth of CD (Theriot et al., 2014). After FMT, beneficial bacteria competed with CD for enteral nutrients and inhibited toxin synthesis or directly caused their death.

### 3.4 Improved intestinal barrier function and regulation of host immunity

FMT restores the biodiversity and richness of intestinal flora, which in turn improves intestinal barrier function, regulates immune responses to resist CDI, and reduces the inflammatory response. The SCFAs produced by the beneficial microbiota after FMT provide energy for intestinal mucosal cells and help reduce intestinal mucosal permeability to repair the intestinal epithelial barrier. Moreover, beneficial bacteria promote the synthesis of mucin 2, antimicrobial peptides, and defensins in epithelial cells and reduce the pH of the intestine to help repair the intestinal chemical barriers in patients with CD (Li et al., 2015). After transplantation, innate immune cells and excited epithelial cells release proinflammatory cytokines and chemical activators. These chemical molecules can activate immune cells, induce the expression of antimicrobial peptides and production of active nitrogen and reactive oxygen species (Madan and Petri, 2012; Solomon, 2013), and initiate the host immune system.

## 4 Relationship between CDI and kidney transplantation

Renal transplantation, which is the most effective treatment for end-stage chronic renal failure, greatly improves the quality of life of patients with end-stage renal disease. The composition of the intestinal flora in patients after renal transplantation is significantly different to that before surgery, which significantly impacts the survival rates of transplanted kidneys. A recent study found that a significant decrease in the diversity of intestinal flora in recipients after renal transplantation was correlated with the occurrence of infectious diarrhea (Casper Swarte et al., 2020). Due to dietary restrictions after renal transplantation and the use of immunosuppressive agents and antibiotics, the original healthy intestinal microbiomes of recipients are destroyed, which reduces their immunity and increases the risk of opportunistic infections. The incidence of CD-associated diarrhea in KT recipients is 1.8–12.4% (Ni and Qi, 2006; Wu et al., 2013), which has increased in recent years. Furthermore, the virulence of the strain and susceptibility of the population are growing, which is currently a key challenge.

CD is present in normal intestinal flora. When the intestinal structure and flora balance are disrupted, CD can transform from a non-invasive state to an invasive state, causing intestinal CDI. For example, the immunosuppressant mycophenolic acid (MPA) (Sandborn et al., 2003), which exhibits the highest diarrhea rate after renal transplantation compared to that of other immunosuppressants, is metabolized by uridine diphosphate glucuronosyltransferase to the inactive state metabolite myeophenolieaeid7-glueuronide (MPAG). Although MPAG has no activity, it is discharged into the small intestines with bile after hepatoenteral circulation and is a precursor of inflammatory factors. It directly stimulates the intestinal mucosa, causing local chronic inflammation, pseudomembranous enteritis, duodenal villus atrophy, and other pathological changes (Weclawlak et al., 2011). Subsequently, normal nonpathogenic CD in the intestines changes to the invasive state and intiates intestinal CDI. After colonizing the intestines, CD releases large amounts of toxins A and B, thereby accelerating intestinal damage (Ma et al., 2021). In addition, approximately 15–25% of antibiotic-associated diarrhea, 50–75% of antibiotic-associated colitis, and 95–100% of pseudomembranous colitis cases are caused by CDI (Bartlett and Gerding, 2008). CDI can cause symptoms such as diarrhea, fever, abdominal pain, and abdominal distension. In the early stages of diarrhea, stools are watery and can later develop into purulent bloody stools. Patients with severe infections can experience watery stools accompanied by dehydration and toxic colitis; furthermore, severe diarrhea can lead to death (Wang et al., 2021). In the 2013 US Bacterial Resistance Threat Report, CD was included at the highest emergency level and replaced methicillin-resistant *Staphylococcus aureus* as the leading cause of hospital-acquired intestinal infections and antibiotic-associated diarrhea (Howell et al., 2003). It has been proposed that when chronic diarrhea occurs after an allogeneic transplantation, opportunistic infections after intestinal dysbacteriosis must be assessed, and the occurrence of CDI should be actively prevented and infections should be treated to restore intestinal function and protect the stability and function of the transplanted kidney (Ma et al., 2021).

## 5 Relationship between FMT and kidney transplantation

### 5.1 Recent advances in FMT for treating CDI-associated diarrhea after renal transplantation

To date, the overall incidence of CDI in renal transplant recipients is 1.8–16.0% (Ni and Qi, 2006; Dubberke and Burdette, 2013; Wu et al., 2013), and the overall cure rate of FMT in solid organ transplant recipients is approximately 79–89% (Kelly et al., 2014). Friedman-Moraco et al. (2014) reported the clinical outcomes of two solid organ transplant recipients (lungs and kidneys) treated with FMT after CDI. The symptoms of both patients initially improved. In the first few weeks after the first FMT, one patient experienced recurrent diarrhea. However, both recipients achieved a lasting treatment response after the second FMT. Gu et al. (2018) elucidated the outcomes of FMT in four patients with refractory diarrhea after renal transplantation between 2014 and 2017. The electrolyte absorption rate of the patients improved after FMT, and the tacrolimus and creatinine concentrations did not significantly change. Lu et al. (2018) retrospectively analyzed the nursing processes of two patients that underwent FMT for post-renal transplantation recurrent diarrhea. After treatment, the symptoms were relieved in both patients. Cao et al. (2021) determined the efficacy and safety of FMT in two patients with post-renal transplantation CDI; both patients were cured without adverse reactions.

The use of FMT for treating CDI-associated diarrhea after renal transplantation is still in the preliminary exploration stages. The screening criteria for related donors, accurate detection of pathogenic bacteria, quality, safety of bacterial solution preparation, understanding of FMT, and standardized operation of the transplantation process need to be further explored (Petrof et al., 2013; Ren et al., 2016). Many medical institutions have tried to use FMT to treat refractory diarrhea after renal transplantation; however, it is unclear if FMT can be used to treat diarrhea after other solid organ transplantations. FMT methods could be improved by developing automatic bacterial separation equipment, standardized laboratory technologies, technical approval, and supervision.

### 5.2 Nursing processes of FMT

#### 5.2.1 Pre-transplantation care

##### 5.2.1.1 Screening and selection of FMT donors

There are two standard approaches for donor selection. The first method is recipient-oriented. This approach is used for autologous or allogeneic transplantations. In the latter, the donors are primarily spouses, family members, or friends of the recipient. The second method includes universal donors and stool banking. Donor screening is the first line of defense to ensure the safety of FMT and minimize the risk of disease transmission. The selection of donor feces should strictly follow questionnaire procedures, interviews, and blood and stool examinations. Currently, there are no universal standard screening criteria (Wang et al., 2019). However, in 2021, the International Fecal Library OpenBiome released the latest comprehensive donor screening criteria (Openbiome, 2021). In 2022, Japanese scholars updated the donor screening criteria by summarizing their own experiences in diagnosis and treatment with FMT (Zhang X. et al., 2022). In the same year, Chinese researchers proposed screening criteria based on location by evaluating 8,483 candidate donors (Zhang S. et al., 2022). Different countries or regions have different regulatory requirements for FMT, and clinicians should adjust the screening criteria for donors according to the regulatory and legal requirements of their region (Wang et al., 2022).

Questionnaires and interviews are used to prevent issues, including those that cannot be detected by blood and stool tests, based on age, body mass index, high-risk sexual behavior, chronic diseases, and history of mental illness. Given the case reports on FMT for treating autism, depression, and other psychological diseases (Doll et al., 2022) and studies on the gut–brain axis, scholars have proposed that good mental health is essential for successful FMT. Therefore, the latest screening standards also include psychological evaluations, such as the self-rating depression scale, self-rating anxiety scale, and Pittsburgh sleep quality assessment (Zhang S. et al., 2022). In addition, Chen et al. (2021) suggested that donors with severe food allergies should be excluded. Furthermore, hematological and serological tests are performed to exclude donors with blood-borne pathogens and systemic organ dysfunction. These tests include whole blood cell count, liver and kidney function, electrolytes, and infectious disease analyses (Wang et al., 2022). Detailed laboratory testing of fecal microorganisms is also performed to assess bacterial abundance and the presence of infectious pathogens (Khan et al., 2018). At present, there is no precise data that indicates that screening criteria other than medical histories and laboratory tests are sufficient for donor selection. Donors who meet these requirements are encouraged to eat foods rich in dietary fiber and fast-sensitive drugs in preparation for collecting high-quality specimens.

Due to the stricter screening criteria for FMT donors, only approximately 10% of donors meet the requirements and are recipient-oriented (Wang et al., 2022). Considering the advantages of easy access and quality assurance, healthy but qualified universal donors or fecal banks unrelated to recipients have become a popular clinical choice. Recently, a universal donor feces bank that accepts regular screening procedures was established for patients who need emergency FMT. China has also established the Chinese Fecal Bacterial Bank (http://fmtbank.org/knowledge/) to achieve the remote emergency rescue and standardization of fecal bacterial preparations and storage (Zhang and Zhang, 2021).

##### 5.2.1.2 Preparation of the recipients

###### 5.2.1.2.1 Psychological nursing

Strong communication between doctors, nurses, and patients is necessary. Before transplantations, the principles, methods, cooperation requirements, and related precautions for FMT should be explained to the patients and their families through pictures, videos, audio recordings, and brochures. In addition, patients should be provided with information about the relative efficacy of the surgery, the surgery itself, and the expected results to eliminate patient anxiety (Xu et al., 2018), build confidence, and obtain written informed consent effectively.

###### 5.2.1.2.2 Antibiotic pretreatment

It is unclear if antibiotic pretreatment before FMT is beneficial. Currently, the most commonly used antibiotic pretreatment in clinical practice is vancomycin (500 mg, twice daily) combined with metronidazole (250 mg, twice daily) for 7–10 days. If antibiotic pretreatment is used, antibiotics must be discontinued 1–2 days before surgery to prevent damage to new flora (Leis et al., 2015).

###### 5.2.1.2.3 Identification of the transplantation routes

Two main routes have been identified for FMT: the upper gastrointestinal tract (oral, nasogastric tube, gastroscopy, gastrostomy, gastroduodenoscopy, nasoduodenal tube, etc.) and the lower digestive tract (retention enema, colonoscopy, colostomy, etc.). Oral capsules, colonoscopies, and enemas have been widely used in clinical transplantation (Kelly et al., 2015). Currently, there is no consensus on which is the best transplantation route as recent studies have shown no significant difference in the clinical efficacy of FMT administered through the upper or lower digestive tracts (Brandt and Aroniadis, 2013). In addition, oral capsules with donor microbiota can reduce the risk of intestinal mucosal injury and reflux pneumonia that are caused by nasointestinal tubes and associated with invasive treatments, such as upper gastrointestinal endoscopies and colonoscopies (Gao et al., 2022). Oral administration is easy and highly accepted by patients. However, the number of bacteria obtained for the process can be limited; therefore, they must be repeatedly obtained to make them more susceptible to gastric acid and reduce the colonization rate (Yu et al., 2020). Retention enemas are more economical and present less surgical risk; however, there are limited retention donor materials, and retention enemas also require multiple treatments. The less invasive methods, such as retention enemas or nasointestinal infusions, may be safer for frail or seriously ill patients undergoing FMT. Thus, the specific needs of the patient, determined by their general situation, lesion location, disease characteristics, convenience, acceptance, and economic costs, must be considered to establish treatment strategies.

###### 5.2.1.2.4 Digestive tract preparation

The transplantation route should be determined based on the condition of the recipient. Given the risk of reflux and aspiration, it is recommended that recipients undergoing FMT through the upper gastrointestinal tract fast for at least 4 h before FMT. Gastrointestinal motility drugs (metoclopramide, 10 mg, intramuscular injection) can also be used the day before the FMT to promote gastric peristalsis and prevent adverse events, such as reflux and vomiting (Singh et al., 2007). Proton pump inhibitors can be administered orally to reduces the interference of the gastric acid environment with the colonization of transplanted microorganisms. For inhibitors that pass through the lower digestive tract, intestinal preparation can be performed, and drugs that inhibit intestinal peristalsis can be used. Regardless of the transplantation route, recipients should undergo large-volume intestinal lavage before transplantation to reduce the resident *Clostridium* population (Brandt and Aroniadis, 2013). A previous study showed that intestinal lavage alters the mucosal adhesion of flora (Harrell et al., 2012), however, whether this change affects the efficacy of FMT has not been confirmed.

#### 5.2.2 Operation and coordination of FMT

Before performing FMT, it is essential to conduct a comprehensive evaluation of the patient’s general condition and provide them with a detailed explanation of the implementation process and collaboration points. (1) FMT via nasoduodenal tube: Patients should remain in a semi-upright position for optimal placement and function of the tube. After catheterization, the catheter is secured with tape using spiral fixation and is properly labeled. The rewarming bacterial solution is extracted using a 50 ml light-resistant syringe and administered to the patient via the nasointestinal tube, out of their line of sight, for 3–5 minutes. Following infusion, the tube is flushed with 10 ml of warm normal saline and the patient is instructed to remain seated or semi-supine for at least 1 h. (2) FMT via esophagogastroduodenoscopy: 30 minutes to 1 hour prior to treatment, 10 mg of metoclopramide is administered intramuscularly, followed by intravenous administration of proton pump inhibitors to inhibit gastric acid secretion and prevent stress ulcers. During the treatment, patients should remain in a left lateral decubitus position with the head of the bed, elevated to 30. Then, the endoscope is inserted into the duodenum, followed by insertion of a catheter through the working channel of the endoscope and injection of bacterial solution. The injection speed must be controlled, and a negative pressure aspirator should be prepared for backup while closely monitoring the patient for potential adverse reactions, such as coughing, nausea, vomiting, and abdominal pain and distension. (3) FMT via retention enema: First, the patient’s position and the depth of the anal canal insertion are determined based on the patient’s condition. Then, the patient is instructed to breathe deeply. (4) FMT via colonoscopy: Patients are instructed to bend their knees in the left lateral decubitus position. Operations should be carried out according to the patient’s feedback. Once the front end of the injection hose appears in the display, the bacterial solution is slowly introduced. The injection speed should be controlled, and the patient’s vital signs should be closely monitored throughout the infusion process while remaining vigilant for any indications of facial or abdominal discomfort or distension.

#### 5.2.3 Post-transplant care

##### 5.2.3.1 Disease observation

Despite current evidence of the safety of FMT, adverse reactions can occur (Xu et al., 2019). Minor adverse reactions include abdominal discomfort, diarrhea, constipation, abdominal stridor, abdominal distension, nausea, vomiting, and spontaneous fever. Serious adverse reactions include endoscopic complications (perforation and bleeding), sedation-related complications (bronchial aspiration), pathogen transmission, and infection (peritonitis and pneumonia) (Wang et al., 2022). Therefore, vital signs should be closely monitored after FMT every 30 min for 2 h and every 4 h thereafter. Nausea, vomiting, abdominal pain, abdominal distension, and diarrhea may be observed. Accurately recording the number, color, volume, and other traits of the stools can guide the retrieval of necessary specimens. Doctors should be consulted if perforation, bleeding, or aspiration occur.

##### 5.2.3.2 Volume and immunosuppressant concentration detection

Severe diarrhea can lead to insufficient blood volumes, electrolyte disorders, and increased serum creatinine levels in patients with CDI after renal transplantation. Therefore, attention should be paid to changes in patient weight, the 24-hour fluid intake and output should be accurately documented, the volume overload of patients should be promptly assessed, and the appropriateness of fluid therapy should be ensured. Severe diarrhea affects the absorption of immunosuppressive agents; therefore, immunosuppressive regimens should be adjusted as needed and the drug concentration, renal function, and electrolyte levels in patients should be monitored.

##### 5.2.3.3 Diet, medication, and body position management

Postoperative fasting should take place for 4 h (Brandt and Aroniadis, 2013), after which patients should on consume liquid food, such as rice soup or porridge. Patients should eat fewer and more meals with light food and gradually transition from a liquid diet to semi-liquid, soft, and general food (Lu et al., 2018). They should avoid consuming crude fiber, spices, stimulating foods, and gas-producing foods, such as radishes or beans. High-protein diets should be avoided. To avoid immune rejection, patients should avoid eating foods and health products that can improve their immune function. The metabolism of immunosuppressants in the body can be affected by grapefruit; therefore, grapefruit should also be avoided (Xu et al., 2018). To ensure the transplanted microbiota colonize, patients should avoid using antibiotics in soon after FMT and should be prescribed immunosuppressive agents. Patients should be advised to comply with the treatment.

Patients undergoing FMT through the upper gastrointestinal tract should assume a sitting or semi-reclining position after transplantation. Their position can be changed after 1 h. Patients treated via the intestinal tract should avoid defecation for 2 h after surgery. They should be instructed to raise their buttocks and perform levator ani exercises to prolong the retention time of bacteria in the intestinal tract and improve the colonization rate.

##### 5.2.3.4 Discharge guidance

Discharge guidance should include: (1) a guide for self-observations of symptoms such as abdominal pain, bloating, nausea, and vomiting; (2) a guide to developing good bowel habits and making stool observations, paying attention to color, character, and quantity; (3) instructions to take immunosuppressive agents in strict accordance with the advice of doctors, with attention to the signs of transplant rejection and infection of the transplanted kidney. Patients should visit the doctor immediately if there are abnormal conditions. Other aspects to consider include: (4) changes in blood biochemical indices, and routine blood tests should be conducted regularly; (5) personal hygiene and infection prevention should be maintained; (6) patients should have scheduled rest and activity and diet management; and (7) patients should be followed-up with regularly.

## 6 Conclusion and outlook

FMT, a rapid technique for repairing the microbiota, has been shown to be more effective than general drug therapy in treating rCDI. It is also considered safe and effective for kidney transplant recipients who are immunocompromised, without increasing the risk of infection or causing obvious complications. However, there remains considerable scope for future research and clinical application of FMT. The intestinal virome is an important component of the human intestinal microecosystem. More and more evidence has demonstrated that the transfer of intestinal virome in FMT is essential for the efficacy of the therapy. Studies have shown that the efficacy and long-term effects of FMT in treating CDI are related to the composition of the donor’s gut virome and the colonization status of the recipient’s gut virome (Qiu et al., 2023). In comparison to the intestinal bacteria, the intestinal virome still harbors numerous unknown mechanisms in disease occurrence, development, and treatment that warrant further investigation. The intricate interplay between the intestinal virome, bacteria, mycobiome, and human immune system constitutes pivotal factors in disease management. Advancements in omics and sequencing technologies will elucidate the complex relationships between intestinal viruses, microecology, and host immunity, underscoring the substantial therapeutic potential of the intestinal virome. Moreover, for infectious diseases caused by pathogenic bacteria, the intestinal virome will also offer personalized targeted treatment options based on precision medicine, thereby reducing reliance on conventional antibiotics and mitigating the emergence of antibiotic resistance, thus expanding the potential for therapeutic interventions. This study had some limitations. First, papers published in less-common languages may have been excluded. Second, “grey literature” was not included, and publications that were not available as full texts were not included, which may have increased the risk of publication bias. These limitations should be addressed in future studies.

## Author contributions

YL: Project administration, Resources, Supervision, Writing – review & editing. YY: Conceptualization, Data curation, Methodology, Writing – original draft. NY: Conceptualization, Investigation, Methodology, Supervision, Writing – review & editing. QW: Data curation, Methodology, Writing – review & editing. JY: Conceptualization, Data curation, Methodology, Writing – review & editing. JG: Data curation, Methodology, Writing – review & editing. HZ: Supervision, Writing – review & editing.
